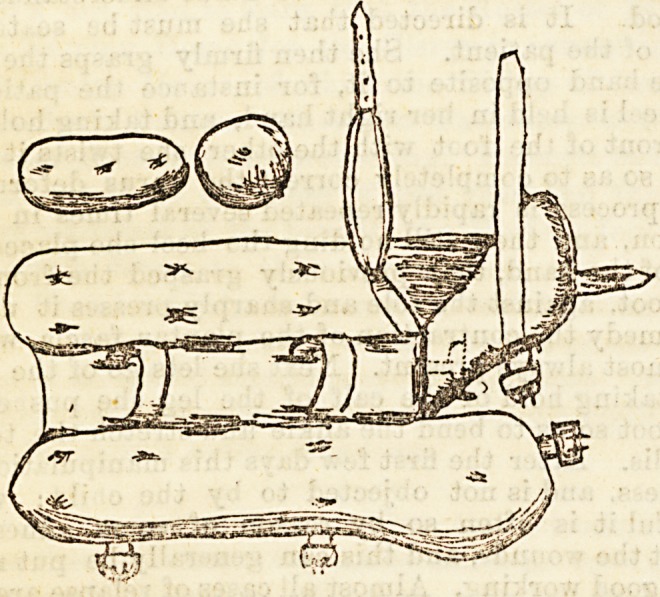# The Treatment of Club Foot

**Published:** 1893-04-15

**Authors:** 


### The Hospital Clinic.

[The Editor will be glad to receive offers of co-operation and contributions from members of the profession. All letters should be addressed to The Editor, The Lodge, Porchester Square, London, W.]

### ROYAL ORTHOPÆDIC HOSPITAL.

### The Treatment of Club Foot.

At the Royal Orthopædic Hospital the opinion is held that all forms of club foot are best treated by subcutaneous division of any tense tendons and fascial and ligamentous bands, followed by correction of the deformity by manipulation, either by means of the hand or by appropriate instruments. It is considered that the severer forms of treatment by removal of portions of the bony framework of the foot are seldom if ever required, and that in all cases the best results can be obtained in the above manner, providing that a sufficient degree of skill is shown in the treatment. The method will be best understood by following the progress of a typical case through all its stages, and then noting peculiarities required in the treatment of other less straightforward cases.

*Treatment of Congenital Equino-varus.*—In this deformity, as is shown in the diagram, the foot is turned inwards and the heel raised, so that if the condition is still present when the child begins to walk the pressure falls upon the side or even upon the upper part of the outer surface of the foot. Treatment is commenced at an early age, if possible within the first few weeks of life, and under these circumstances a good result is regarded as certain. At the first sitting the anterior and posterior tibial tendons are divided in the usual way, the punctures being closed by simple pieces of lint fixed with strapping. The foot is twisted to make sure that the tendons have been thoroughly divided, and it is then fixed by a splint in the position that it most readily assumes, no attempt being made at this time to force it into a good position. The splint used is made of a piece of ordinary barrel-hoop iron, padded with wadding. Its advantages are that it is cheap and light, that it can be bent into any necessary shape, and that, being narrow, it takes a good grip of the foot. Four days after the operation the splint is removed without the pad of lint over the wound being disturbed. The foot is gently worked so as to bring it into a good position, and it is then replaced upon the splint, which is bent so as to fit the new and improved condition. This process is repeated every two or three days for about a fortnight, by which time the varus deformity is generally entirely overcome, and the foot can, without any undue force, be turned outwards instead of inwards. If there is any tightness of the plantar fascia, it is remedied at this stage of the treatment by division of the contracted band while an assistant puts it "on the stretch." It is afterwards more completely relaxed by frequent manipulation with the hand. The tendo achillis is now divided in the usual way, and the foot fixed on a splint, just as in the case of the tibial tendons. Every two or three days the splint is removed, and the foot gently manipulated. In the course of ten days to a fortnight the heel can generally be brought right down to its normal position. At this stage the iron splint is replaced by a simple permanent instrument called variously a night shoe or a simple varus splint.

As can be seen from the diagram, this splint consists of a flat sole-plate fixed at right angles to a back piece which comes nearly to the knee, and is rounded so as to accommodate itself to the shape of the calf. At the outer edge of the sole plate there is a ridge to which a strap is fixed. The instrument is applied by pushing the foot into it so that the heel goes well down into the hole cut for it at the angle. It is retained in position by a strap passing over the ankle, and the leg is fixed by a band passing round it. The varus is then corrected by drawing the foot out by means of the strap fastened to the ridge. This instrument is of great value and much depends on its proper application. For a couple of weeks the shoe is removed every second morning, and the foot well worked; after that the foot is worked every morning before the application of the instrument. As much of the success of the treatment depends upon the way in which the manipulation is carried out, great care is taken to see that the mother or nurse understands the method. It is directed that she must be seated in front of the patient. She then firmly grasps the heel in the hand opposite to it, for instance the patient's left heel is held in her right hand, and taking hold of the front of the foot with the other she twists it outward so as to completely correct the varus deformity. This process is rapidly repeated several times in succession, and then still holding the heel she places the flat of the hand, that previously grasped the front of the foot, against the sole and sharply presses it up so to remedy the contraction of the plantar fascia, which is almost always present. Next she lets go of the heel, and taking hold of the calf of the leg she pushes up the foot so as to bend the ankle and stretch the tendo achillis. After the first few days this manipulation is painless, and is not objected to by the child; when painful it is often so by reason of some adhesions about the wounds, and this can generally be put right by a good working. Almost all cases of relapse are due to the fact that the parents or nurse have neglected to continue the working of the foot. Although there is little risk of overdoing it, yet it is well to take care not to overstretch the plantar fascia, which would give rise to flat foot, nor the tendo achillis, which would cause talipes calcaneus, that is to say the child would walk on its heels.

Night Shoe.

When the child is beginning to walk it must be supplied with proper instruments. These consist of a band round the waist, with an iron coming down the outside of the leg to the knee. It is there connected to a ring encircling the leg just below the joint, and then runs down the inside of the leg past the ankle, to end in a tongue inserted between the layers of the sole. The instrument is hinged at the hip, knee, and ankle. It is necessary that, from the knee down, the iron should run down the inside of the leg; since were it on the outside any attempt to turn the foot outwards by its means would draw the tongue from its socket in the sole, whereas since the point at which you wish the torsion outwards to occur is at the back of the foot, any twisting of the iron so as to turn the foot out only tends to press the tongue more firmly into its socket. This is of especial importance, since in these cases all the leg, but especially from the knee downwards, is turned inwards, and considerable force is needed to keep it turned out while the child is walking. To remedy this irons are sometimes ordered to be worn during the daytime for a considerable period before the child can walk.

The deformity having been corrected in the way described irons are worn during the day, and the night shoe at night. In addition, the foot is frequently worked in the way that has been described. All fear of relapse is thus avoided, and after a few years the foot having become "set" in the natural position the irons are finally discarded.

In the treatment of older cases than those spoken of above the method is the same. As perfect a result, however, cannot be expected, and a good one can only be obtained by care, patience, and skill.

Relapsed cases: These are cases in which treatment has been attempted without success, or in which the treatment having been successful the deformity has been allowed to return from want of attention. They are always found to be more difficult to treat than cases that have not been interfered with, and in some of them it is found to be impossible to obtain a good result. Treatment is pursued along the lines described above. The foot must be frequently worked, if necessary under chloroform. A strong hand is of great advantage, as it often enables the operator in a moment to overcome a difficulty that would have required weeks to correct by means of an ordinary instrument. If necessary a Scarpa's shoe is applied, or else a modification of it, called a pad instrument.

This instrument was invented by Mr. Henry Baker who claims for it that it grasps the leg more firmly than the Scarpa's shoe, and that a better purchase can, therefore, be obtained in correcting the varus. Furthermore, by the aid of the pads, from which it derives its name, the point of pressure can be changed day by day, and thereby the risk of the formation of sores is lessened.

The instrument, as can be seen from the diagram, consists of a leg-piece, to which, by means of a rack and pinion joint, a sole plate is fixed so that its motion is the same as that of the ankle joint. To the outer side of the sole plate near the front a strap is fixed. The leg-piece is formed of a central plate and two wings. The central plate runs up the back of the leg from the sole plate almost to the knee. The wings are attached to it by hinges. They project above slightly higher than the central piece, so as to grip the leg. Below they descend considerably lower than the sole plate, so that they can be fastened together by a strap beneath it. The parts of the leg-piece are held firmly together by two straps, which encircle them and the leg together. The instrument is applied just like a Scarpa's shoe. The heel having been secured in place with a strap, the leg is fixed to the instrument, and then the requisite flexion of the ankle having been obtained by means of the screw, the front of the foot is drawn out by means of the strap attached to the sole plate. Whatever form of instrument is used, care is taken to remove it every day, and the limb is washed, dried, and dusted with starch powder. In this way it is found that even great pressure can be endured for long periods without injury.

A point that is found of considerable advantage in the instrumental treatment of young children is to put the instrument into the required form, and then with the hand gradually mould the foot to fit it. It is found that in this way a greater advance can be made with safety and comfort than is possible, if, as in the case of older patients, the instrument is applied to the foot, and then by means of the screw mechanism is forced into the desired position.

*Paralytic Club Foot.*—Treatment is pursued as in the congenital cases. It is, of course, hopeless to expect the same good result since, although the deformity can be corrected, it is impossible to restore strength to the wasted muscles, or to cause a natural growth of the shrunken limb. A point of some interest is that the paralytic cases are more varied in nature than the congenital ones; for instance, talipes equinus is one of the commonest forms of paralytic deformity of the foot, whereas uncomplicated with inward distortion it is very rare as a congenital defect. In the treatment, great care is exercised to prevent overstretching of the tendons that have been divided; and in order to ensure this, the splints are kept on much longer than is considered necessary in the congenital cases. Again, since the circulation in the affected limbs is always very weak, they are kept well wrapped up, and in order to prevent the formation of sores undue pressure is carefully avoided.

In the attempt to restore strength to the wasted muscles, massage is regarded as of even greater value than electricity, and by its continued use good results are occasionally obtained even in somewhat severe cases. When ordering instruments, the attempt is made to supply the place of weak and paralysed muscles by the use of rubber springs, and by limiting the movement of the joints to take the strain caused by the weight of the body off the paralysed parts.

*Spastic Cases.*—These are cases in which the deformity is kept up and caused by morbid contraction of muscles. They are generally the result of disease of the spinal cord. In few of them can any permanent good result be expected, but relief is often afforded by division of the tendons of the affected muscles, and the application of instruments to prevent the return of the deformity.

## Figures and Tables

**Figure f1:**
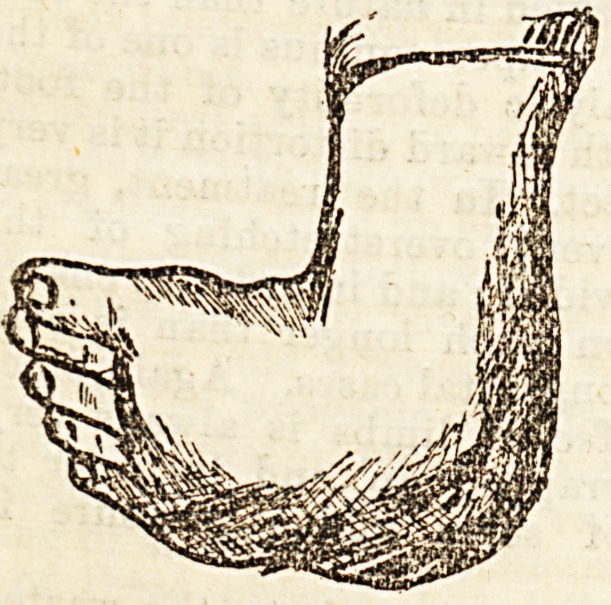


**Figure f2:**
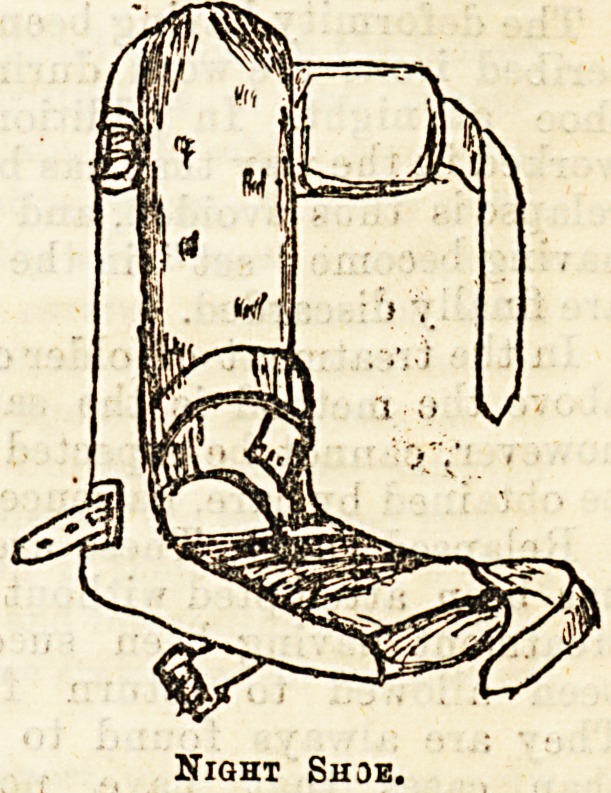


**Figure f3:**